# Prevalence and severity of anxiety symptoms among family members of nyaope users in Tshwane, South Africa

**DOI:** 10.4102/hsag.v28i0.2083

**Published:** 2023-09-29

**Authors:** Kebogile E. Mokwena, Maphuti C. Madiga

**Affiliations:** 1 Department of Public Health, School of Healthcare Sciences, Sefako Makgatho Health Sciences University, Pretoria, South Africa

**Keywords:** General Anxiety Disorder, mental health, family members, nyaope, Tshwane, South Africa

## Abstract

**Background:**

Nyaope is a strongly addictive novel psychoactive substance that is commonly used in predominantly black townships in South Africa. The undesired behaviours of the users result in family members developing mental health challenges. Nyaope users often commit petty crimes, including stealing from families and neighbours.

**Aim:**

The aim of this study was to quantify anxiety symptoms among family members of nyaope users in Tshwane, South Africa.

**Setting:**

Data were collected from nine townships within Tshwane Metropolitan Municipality.

**Methods:**

The quantitative cross-sectional survey used the General Anxiety Disorder (GAD-7) tool to quantify anxiety symptoms, and a questionnaire was used to collect sociodemographic data from a sample of 390 participants.

**Results:**

The ages of the participants ranged from 18 years to 87 years, with a mean of 47 years. The mothers to the nyaope users were the biggest group at 35% (*n* = 138). The prevalence of anxiety symptoms was 73% (*n* = 286) of the total sample and ranged from mild (41.8, *n* = 163), moderate (14.62, *n* = 57) and severe (16.92%, *n* = 66). The Pearson chi-square test identified significant associations between anxiety symptoms and the gender of the participant (*p* = 0.001), the age of the nyaope user and the period of nyaope use (*p* = 0.008). Multivariate regression model indicated gender and place of residence as a significant variable in the development of anxiety symptoms (*p* = 0.01).

**Conclusion:**

Nyaope use is a risk factor for the development of anxiety for family members of nyaope users with the highest proportion reporting mild symptoms.

**Contribution:**

There is a need to develop interventions for mental health support for families of nyaope users.

## Introduction

As the global disease patterns continue to lean towards noncommunicable diseases, both substance abuse and mental disorders contribute to a significant proportion of those cases. The last decade has seen a significant increase in the prevalence of substance abuse, especially in developing countries (Moradi, Lavasani & Dejman 2019). In South Africa, the increase in substance abuse occurs in an environment of inadequate prevention strategies, lack of treatment services, as well as insurmountable barriers to treatment, which includes cost, transport and availability of related resources (Pasche & Myers 2012).

Substance abuse is a leading social problem for the well-being of both the users and their families (Frans 2020). Because substance abuse patterns tend to be determined geographically, this mental challenge extends from family to community. The high prevalence of substance abuse in South Africa implies related high prevalence of family and community mental illness, which does not enjoy the attention it deserves. Moreover, family members and caregivers of patients living with mental illness, which includes substance abuse, also experience stigma and have their own set of mental illness. Continuous efforts to withhold the patient’s diagnosis further contribute to mental stress, which requires mental health attention for the caregivers or family members of the patient (Monnapula-Mazabane & Petersen 2021).

Literature about the mental health consequences of psychoactive substances mainly focuses on the impact of the psychoactive substances on the user’s social, physical and mental health, but less so on the health impacts of family members of the user. This limited user focus includes prevention and aftercare programmes, despite acknowledgement that family members are impacted by substance abuse of any member of their household. Family education has been identified as one of the most important substance abuse prevention strategies (Moradi et al. 2019), hence the need to consider, acknowledge and include the family in substance abuse studies and interventions. There is therefore a need to pay attention to the impact of substance use disorders on the family members (Lander, Howsare & Byrne 2013).

Substance abuse is associated with a range of criminal behaviours and violence perpetrated by the user (Etta & Ojedokun 2017), which affects the mental well-being of family members. Although the violence can be directed at anyone, family members are at increased risk because of frequent interactions with the substance user. In South Africa, where services for substance use disorders are limited, and both human resources and evidence-based policy and service planning frameworks are limited (Pasche & Myers 2012), the impact is significant and renders the victims powerless and hopeless.

The negative social impact of substance abuse often extends to neighbours, friends and co-workers (Rosenbloom et al. 2010). While mental health affectations occur across substance abuse in general, specific behaviours and the intensity of impact tend to be aligned to certain substances. In particular, literature reports associations between substance use disorders in the patients’ families, with depression and generalised anxiety being the most common (Solati & Hasanpour-Dehkordi 2017). In the case of nyaope use, the impact on the family seems to be particularly intense, as nyaope use interferes with family trust and by extension to relationships, as nyaope users are increasingly isolated from their other social structures.

## Nyaope, the novel psychoactive substance of abuse in South Africa

Nyaope is a novel psychoactive substance (NPS), which emerged in Tshwane Metropolitan Municipality in the late 1990s and early 2000s (Masombuka 2013; Mokwena & Morojele 2014). Nyaope is highly addictive, and most of the users find it difficult to quit, making it a lifelong problem (Masombuka 2013). The users can easily be recognised as they assemble in public places such as parks, taxi ranks and shopping malls. They show slow speech and movements and are known for poor hygiene. As a result, nyaope use is easily identified, especially by family members who can notice trends and behaviours well known to be traits of the use of this cocktail drug.

Nyaope is easily accessible because of the relatively low price of between R35.00 and R50.00 (which can be as low as $2.00 per fix) by many dealers, as well as by poor law enforcement efforts in predominantly black communities (Masombuka & Qalinge 2020). These factors enable easy access to many vulnerable young people, including primary school learners. Nyaope is commonly used among people of low socioeconomic status, and not adequate attention is paid to its individual and even community impact (Mokwena & Huma 2014). This has resulted in affected communities being left to deal with a range of social and mental consequences. Users are almost all unemployed, have dropped out of school or left their employment, which drives them to steal to feed their habit. Many of the users live in the street in the company of other users, and they have formed communities that support each other in their quest to get the next fix. This increases anxiety levels among families and community members.

While anxiety has been reported among family members of substance abusers (Ólafsdóttir, Hrafnsdóttir & Orjasniemi 2018; Vorspan et al. 2015), anecdotal evidence suggests that anxiety levels among family members of nyaope users may be higher. This is because of the addictive nature of the drug, poor prognosis, the habit of stealing from the community, including family, and this resulted in nyaope use taking over the lives of the users. However, attention and literature on the impact of nyaope on the families and communities of the users is limited.

Rehabilitation facilities for drug users are scarce, and even the few available ones do not have a custom-made treatment programme that is specific to nyaope. This has resulted in very high relapse rates, which makes people believe that rehabilitation for nyaope users is not helpful, and this view influences help-seeking behaviour negatively. Because of the socioeconomic status of the users, most depend on public facilities, as they cannot afford private facilities (Monyakane 2018). The few publicly funded facilities are always full, and users typically apply and are placed on a waiting list before they can be admitted. Often the waiting period before being allocated in-house treatment lasts between 6 months and up to 2 years. During that time, the users are lost in the system or lose interest for rehabilitation as the intensity of the addiction increases, which has mental health impact on their families.

Evidence-based practice motivates for the aftercare and reintegration services following in-house rehabilitation of substance abusers (Gonzales-Castaneda et al. 2022). However, in South Africa, such services are not systematically enabled, as they are met with challenges that include unclear policies and scarce resources, which form barriers at all levels of service provision (Mpanza, Govender & Voce 2021). For most users of nyaope, such services are non-existent, which puts demands on the family who have to deal with a situation where the needed care is not available (Mahlangu & Geyer 2018). The combination of situations regarding nyaope use renders the family members to be at risk of a range of mental challenges. Therefore, the purpose of this study was to quantify the prevalence and the severity of anxiety symptoms, as well as explore associated factors, among family members of nyaope users in Tshwane Metropolitan Municipality in South Africa. The data were collected simultaneously with a study to screen for depression symptoms among the same sample, which was published separately (Madiga & Mokwena 2022).

## The need and benefits of screening for anxiety in public health practice

Screening, one of the most effective health promotion interventions in public health, is commonly used to enable both the delivery of early interventions and treatment services of those in need (Babor, Del Boca & Bray 2017). Its main benefit is that it enables the identification of undiagnosed disorders before complications occur and thus reduction of treatment costs by enabling early treatment. Screening has therefore been identified as a cost-effective strategy for promoting population health, as well as a strategy to implement integrated health systems (Tariq et al. 2009). Screening is particularly recommended for mental health conditions in the population, as it can assist to estimate the burden of these disorders (Weist et al. 2007).

In the context of anxiety, one of the two most common mental disorders globally, screening is important for anxiety because, although prevalent, its estimated burden in South Africa is not known. Screening is also considered to be an effective tool to protect and bolster prevention services (Martin-Moreno et al. 2012) and should therefore be strengthened in public health services of every country.

Several countries have implemented a policy of screening for mental disorders at population level and primary healthcare settings and regarded this as a cornerstone of the agenda to improve mental health (Gilbody et al. 2006). Screening for anxiety in South Africa will benefit the patients and the public health system, as it will assist in estimating its burden, and planning for appropriate levels of human resources, as one of the strategies to prepare for the envisaged National Health Insurance (NHI).

## Purpose of the study

The purpose of the study was to quantify anxiety levels among family members of nyaope users. The article is part of a broader mixed-method study on mental disorders among family members of nyaope users, and a part of the broader study, which focused on depression, was previously published (Madiga & Mokwena 2022).

### Design

The study used a quantitative cross-sectional design to use the General Anxiety Disorder (GAD-7) tool to screen for the presence and severity of anxiety symptoms among family members of nyaope users.

### Research setting

The study was conducted in nine townships of predominantly black people in the Tshwane Metropolitan Municipality, in the province of Gauteng, South Africa. These townships were Soshanguve, Mamelodi, Ga-Rankuwa, Hammanskraal, Winterveldt, Atteridgeville, Pretoria North, Mabopane and Olievenhoutbosch. Nyaope use is rife in the city and surrounding areas, and anecdotal evidence suggests that in the early 1990s, nyaope use spread from these townships to other areas of the country.

### Population of the study

As a result of the high prevalence of nyaope use, various affected townships in Tshwane have seen the establishment of non-governmental organisations (NGOs) that offer a range of support services for nyaope users. Services commonly offered include providing meals for the nyaope users, linking them to social workers and social services, with the aim of getting them to rehabilitation services, and providing a platform for daycare counselling services both before and after completing a rehabilitation programme. The population in this study consisted of family members of nyaope users who access the services of such NGOs in the identified townships.

### Recruitment

The researcher identified and approached the various NGOs in the townships and requested them to allow access to the nyaope users in their database. Depending on the availability of the users at a particular time, the purpose of the study was explained either in a group session or on an individual basis. The users were requested to provide contact information about their families, who were contacted and requested to participate in the study. In addition, the snowball recruitment technique, which entails asking family members who have participated in the study to inform the researcher or research assistants about other families who have a relative who uses nyaope, was used to recruit additional participants. The research assistants were assigned to specific townships, to repeat the process until the minimum sample size was reached.

### Sampling

Partners, parents, siblings or any relative who share their home with someone who uses nyaope, were aged 18 years or older, and were able and willing to provide informed consent at the time of data collection were included in the study.

### Sample size

The Raosoft sample size calculator (Raosoft 2009) was used to calculate the sample size. The use of the sample size calculator indicates the use of a population size of 20 000 if the population size is not known, as is the case with the population size of nyaope users in Tshwane. The minimum sample size of 377 for the study was determined by allocating one family member for each nyaope user, using a 5% margin of error, 95% confidence interval level and 50% response distribution. Ultimately, a sample size of 390 was used.

### Data collection tool

The GAD-7 tool was used to assess the presence and severity of anxiety symptoms. This tool has enjoyed global use for the screening of anxiety in both clinical settings and population surveys. It is a validated and standardised tool with a high sensitivity and specificity and displays stability at repeated measurements with a Cronbach’s alpha coefficient value of 0.91 (Delgadillo et al. 2012). The tool was also reported to be appropriate for use in multicultural and heterogeneous populations (Micoulaud-Franchi et al. 2016; Sousa et al. 2015). The GAD-7 can also be used to monitor change in symptoms over time and was thus identified as appropriate for use for this study. A quantitative questionnaire was used to collect sociodemographic data.

### Pilot study

A pilot study was conducted by the researcher and research assistant to assess the feasibility of the study in a home setting. The accuracy of the tool was established because the GAD-7 screening tool was previously professionally translated into all the indigenous languages of South Africa for a bigger national study to estimate the burden of anxiety among groups of people in South Africa. The purpose of the pilot study was therefore to assess the feasibility of the study in a home setting, and the pilot study included partners, siblings and parents, to enable the researcher to achieve the purposes of the pilot study for different members of a family. The pilot study confirmed feasibility of the use of the tools, and there was no need to change the data collection questionnaires.

### Data collection

Data were collected by the researcher and research assistants between November 2020 and September 2021. On the day of data collection, the adult person or head of the household, who was confirmed to be a partner, parent, sibling or any relative of the nyaope user, was requested to participate. In cases where there was more than one potential participant, the family was requested to decide who would participate. The potential participant was then given a brief explanation about the study and was requested to participate. If they agreed, they were given an opportunity to ask questions or seek clarifications, which was followed by a request to provide informed consent by signing the informed consent form. The majority of the participants (85.4%, *n* = 333) filled in the questionnaire themselves, while 14.6% (*n* = 57) needed the data collectors to fill the questionnaire for them. Data collection started with the administration of the demographic questionnaire to collect sociodemographic data of the participants, data on their family members who use nyaope and the administration of the GAD-7 screening tool. The language used for data collection was according to the preference of the participant.

### Data analysis

The data were analysed by the first author, with the assistance from the second author. The first author received training on data analysis as part of her training for doctoral studies, and the second author was the supervisor. Stata version 14 was used for both descriptive and inferential analyses. The sociodemographic variables were analysed using descriptive statistics. The presence and severity of anxiety symptoms was determined by the GAD-7 scores, which were categorised as *not anxious* for scores of 4 and below, and *anxious* for scores of 5 and above. The category of *anxious* was further categorised as mild (scores of 5–9), moderate (scores of 10–14) and severe (scores of 15 and above).

The ages of the participants were categorised as younger than 50 years and 50 years above. The ages of the nyaope users were categorised as 30 years and below, and 31 years and above. The number of years of nyaope use was categorised into 10 years and below, and 11 years and above. As needed, categorical variables were encoded to create numerical codes for them to enable the performance of correlation with the categories of anxiety symptoms.

The Pearson chi-square test was used to measure the strength of association between generalised anxiety disorder (GAD) categories and a range of sociodemographic variables. Multiple logistic regression analysis was conducted for the sociodemographic variables that were statistically significantly associated with GAD, using a *p*-value of 0.05.

### Ethical considerations

The proposal to conduct the study obtained ethics clearance from Sefako Makgatho Health Sciences University Ethics Committee (SMUREC/H/270/2019). All the research assistants received professional training on the use of the screening tool, as well as the ethics of research, which included the confidentiality and storage of the collected data. The filled-in questionnaires were stored in a locked cupboard. All the participants provided written informed consent before they participated.

## Results

### Characteristics of the participants

A total of 390 participants consisted of 78% (*n* = 304) females and 22% (*n* = 86) males, with their ages ranging from 18 to 87 years, with a mean of 47 years (standard deviation [SD] = 16.78258). The majority were mothers to the nyaope users, followed by sisters. The rest of the relationships of the participants with the nyaope user are reflected in Table 1. Table 2 shows the township of residence as well as employment and marital status of the participants.

**TABLE 1:** The relationship of the participants with the nyaope user.

Relationship to nyaope user	Frequency	Percentage (%)
Mother	138	35.38
Sister	91	23.33
Brother	46	11.79
Father	27	6.92
Grandmother	25	6.41
Other	23	5.90
Aunt	19	4.87
Uncle	13	3.33
Grandfather	8	2.05
**Total**	**390**	**100.00**

**TABLE 2:** Township of residence, employment and marital status of the participants.

Variable	Frequency	Percentage (%)
**Township of residence**		
Soshanguve	97	24.89
Ga-Rankuwa	94	24.10
Mamelodi	80	20.51
Hammanskraal	75	19.23
Winterveldt	21	5.38
Atteridgeville	9	2.31
Pretoria North	6	1.54
Mabopane	5	1.28
Olievenhoutbosch	3	0.77
Total	390	100.00
**Employment status**		
Unemployed	180	46.15
Employed	104	26.67
Pensioner	95	24.36
Student	10	2.56
Missing	1	0.26
Total	390	100.00
**Education level**		
No formal education	14	3.59
Primary	80	20.51
Secondary	242	62.05
Tertiary	52	13.33
Missing	2	0.51
Total	390	100.00
**Marital status**		
Single	218	55.90
Married	99	25.38
Widowed	60	15.38
Divorced	13	3.33
Total	390	100.00

### Characteristics of the nyaope users

Information about the nyaope users was collected from their family members, who were participants in the study. The ages of the users ranged from 17 years to 55 years, with a mean age of 30 years, with the majority (93%, *n* = 363) being male. The majority (98.7%, *n* = 385) were single and lived in family settings (*n* = 339, 86.92%), compared with 13.08% (*n* = 51) who lived in the street. Table 3 shows other sociodemographic characteristics of the nyaope users.

**TABLE 3:** Sociodemographic characteristics of the nyaope users.

Variable	Frequency	Percentage (%)
**Gender distribution**		
Males	363	93.08
Females	27	6.92
**Ages**		
20 years and below	23	5.90
21–25 years	43	11.03
26–30 years	145	37.18
31–35 years	100	25.64
36–40 years	58	14.87
41 years and above	21	5.38
**Highest education achieved**		
Primary	32	8.21
Secondary	341	87.44
Tertiary	14	3.59
Missing	3	0.77
**Employment status**		
Unemployed	357	91.54
Employed	29	7.44
Student	4	1.03
**Marital status**		
Married	5	1.28
Single	385	98.72

### Information related to nyaope use

The period of nyaope use ranged from 1 to 21 years, with a mean of 9 years (SD = 4.695174). The majority (73.86%, *n* = 288) who used nyaope for the longest period were between 25 years and 35 years of age. The majority (60%) had accessed some rehabilitation services but had relapsed. Ga-Rankuwa, Hammanskraal, Soshanguve and Mamelodi had the highest number of those who have been using nyaope for more than 10 years, and the 21–40 years age category had the longest duration of nyaope use. Table 4 shows nyaope-related information of the users.

**TABLE 4:** Information related to nyaope use.

Variable	Frequency	Percentage (%)
**Age group**		
20 years and below	23	5.90
21–25 years	43	11.03
26–30 years	145	37.18
31–35 years	100	25.64
36–40 years	58	14.87
41 years and above	21	5.38
Total	390	100.00
**History of being arrested**		
No	197	50.51
Yes	193	49.49
Total	390	100.00
**Attended rehabilitation services**		
Never	153	39.23
1–3 times	219	56.15
More than 3 times	18	4.06
Total	390	100.00

### Prevalence and severity of anxiety symptoms among family members of nyaope users

The GAD scores ranged from 0 to 21, with a mean of 8. The prevalence of anxiety symptoms, as indicated by GAD scores of 5 and above, was 73.3% (*n* = 286), as reflected in Table 5.

**TABLE 5:** Categories of presence and severity of anxiety symptoms.

GAD-7 score range	Category of symptoms	Frequency	Percentage of sample (%)	Frequency of positive anxiety	Percentage of positive anxiety (%)
0–4	Little or none	107	27.44	Na	Na
5–9	Mild	160	41.03	163	57
10–14	Moderate	57	14.62	57	20
15 and above	Severe	66	16.92	66	23
**Total**	**-**	**390**	**100.00**	**286**	**100.00**

Na, note applicable.

The majority 57% (*n* = 163) of those who tested positive for anxiety symptoms had mild symptoms.

### The impact of anxiety symptoms on their daily activities

Table 6 shows the participants’ responses regarding the extent to which anxiety symptoms make it difficult for them to perform daily activities.

**TABLE 6:** Experience of difficulty in relation to anxiety symptoms.

Extent of difficulty	Frequency	Percentage (%)
Not difficult	154	39.49
Some difficulty	168	43.08
Very difficult	49	12.56
Extremely difficult	19	4.87
**Total**	**390**	**100**

The majority, 60.5% (*n* = 236), reported difficulties in performing daily activities.

### Factors associated with the development of anxiety symptoms

The Pearson chi-square test was conducted to explore associations between GAD categories and sociodemographic variables, using a *p*-value of 0.05. The gender of the participant and the age of the user were found to be significantly associated with the development of anxiety symptoms at *p*-values of 0.001 and 0.008, respectively, as reflected in Table 7.

**TABLE 7:** Variables associated with the development of anxiety symptoms.

Variable	*p*-value
Area or location or place of residence	0.088
Age of the participant	0.310
Gender of the participant	0.001
Language of the participant	0.117
Religion of the participant	0.611
Relationship with nyaope user	0.071
Marital status of the participant	0.845
Highest education of the participant	0.513
Employment status of the participant	0.512
Age of the user	**0.008**
Gender of the user	0.928
Number of years using nyaope	0.052
Marital status of the user	0.497
Highest education of the user	0.987
Employment status of the user	0.612
Times admitted for rehabilitation	0.681
Lives at home	0.135
Ever been arrested	0.737

Multivariate logistic regression analysis was conducted to determine the association between anxiety symptoms and gender of the participants, as well as the age of the user (*p* = 0.05), and only gender of the participant was significantly associated with developing anxiety symptoms.

## Discussion

A few qualitative studies have reported on mental health problems of nyaope use on family members (Masombuka 2013; Mokwena & Makuwerere, 2021), but as far as the authors know, this is the first study to quantify anxiety symptoms among these members. This is therefore the first study to contribute to the estimation of the burden of mental disorders that are associated with the use of nyaope in South Africa. The very high anxiety prevalence of 73% is concerning, especially considering that it occurs among people who lack resources for the diagnosis and treatment of mental health challenges.

The characteristics of the nyaope users were similar to other studies, which profiled the users as mainly male, single and unemployed (Fernandes & Mokwena 2020; Mokwena & Huma 2014; Njuho & Davids 2010), and these factors are often sources of anxiety among family members. The long period of nyaope use confirms that quitting the use of nyaope is difficult, and that users often spend many years trapped in their situation. The wide age range of the users is of concern, as it suggests that users who are in their late 40s and early 50s initiated the use of nyaope when they were much younger and have not been able to quit. The high proportion of users between the ages of 21 years and 40 years confirms the severe addictive nature of nyaope that renders the users unable to quit, and those who initiated it in their teens continue its use into adulthood, which translates to chronic anxiety among family members. The criminality associated with nyaope use is confirmed by the fact that 49.5% of the users have been arrested, and this may be contributing to the anxiety among their family members.

Some variables, although not statistically significant, were strongly associated with the development of anxiety symptoms, and these include the place of residence, which is supported by previous reports that nyaope use is rife in specific areas, which typically are of low socioeconomic status, have high unemployment rates and lack recreational facilities (Masombuka & Qalinge 2020; Mokwena & Morojele 2014). The relationship of the participant with the nyaope user was also strongly associated with the development of anxiety symptoms, which can be explained by the majority of women participants in this study.

The high prevalence of anxiety symptoms among family members attests to the high prevalence of undiagnosed anxiety in the community and contributes to the estimation of this disorder in the population. This also confirms the need to acknowledge that nyaope brings unique challenges to population mental health in South Africa, as well as the need for increased and specific promotion of mental health (Mokwena 2015). However, such services are almost nonexistent in the research settings of this study.

The gender of the participant was significantly associated with the development of anxiety symptoms, at both levels of analysis, which confirms the findings of other studies which found that the prevalence and levels of anxiety are higher among women (Asher, Asnaani & Aderka 2017; Grenier et al. 2019; Seo et al. 2017). This finding is significant because of the phenomenon of absent fathers, which leaves the mothers to deal with the raising of children on their own. The finding that the relationship of the participant to the nyaope user is significantly associated with the development of anxiety symptoms is aligned with other studies, which reported that mothers of adolescents who use substances experience higher levels of anxiety (Di Sarno et al. 2021). These mothers thus need specific interventions to support their needs as they struggle with the use of substances by their children (Groenewald & Bhana 2016). However, this is the first study of determining anxiety among family members of nyaope users. The finding that the period of nyaope use is significantly associated with the development of anxiety symptoms can be explained by the long periods to which the family is subjected to criminality and other unbecoming behaviours of the nyaope user.

Several studies have reported mental distress among family members of substance abusers (Di Sarno et al. 2021; Dostanic et al. 2022; Mokwena & Makuwerere 2021), and that many of the family members found shelter in their workplaces as their homes become unbearable (Ólafsdóttir, Orjasniemi & Hrafnsdóttir 2020). However, because the majority of participants in this study are unemployed, they do not benefit from the shelter of the workplace from their constant emotional and mental assaults that are perpetrated by the actions or behaviours of their substance-using relatives, thus facing constant exposure to situations that are risk factors for anxiety.

A potential additional contributor to anxiety may be the stigma associated with nyaope use, which has been reported by both the nyaope users and their families (Bala & Kang’ethe 2021; Lefoka & Netangaheni 2021), and real or perceived stigma has been linked to poorer mental health (Birtel, Wood & Kempa 2016). On the contrary, social support has been reported to be associated with greater mental health status. However, there is dearth of programmes that offer such social support in these communities, which even lack most of the basic social and health services.

## Conclusion

The study has identified high prevalence of anxiety symptoms among family members of nyaope users, with the majority reporting mild symptoms, followed by those who reported severe and moderate symptoms. The development of these symptoms, which may be explained by the behaviours of the nyaope users, include their safety concerns, as well as criminal activities, especially stealing from their families in order to sell the loot to feed their habits. Because of the difficulty of quitting nyaope use, the families experience long exposures to nyaope use by their family members and are thus exposed to long periods of anxiety triggers. There is therefore an urgent need to develop and implement mental health support interventions for family members of nyaope users, while, on the other hand, solutions to address the prevalence of nyaope use in communities continue to be explored.

## Summary of major findings of the study

The study quantified the presence and severity of anxiety for family members of nyaope users, and that female members of nyaope users are at increased risk of anxiety. Although the development of anxiety among family members was previously reported for substance use in general and nyaope use, as far as the authors are aware, this study was the first to quantify such anxiety levels for nyaope use.

## Limitations

A limitation of the study was that because the recruitment of the participants was influenced by the variation in the cooperation of the NGOs in linking the research team and families of nyaope users, it resulted in unequal proportion of participants from the different areas.
